# Towards Improvement of Heatwave Warnings for Older Adults: The Case of Queensland Australia

**DOI:** 10.1177/21501319241286584

**Published:** 2024-11-04

**Authors:** Mehak Oberai, Steven Baker, Aaron J. E. Bach, Connor Forbes, Ella Jackman, Sebastian Binnewies, Zhiwei Xu, Sarah Cunningham, Son Nghiem, Dung Phung, Shannon Rutherford

**Affiliations:** 1 School of Medicine and Dentistry, Griffith University, Gold Coast, AU-QLD, Australia; 2 School of Health Sciences and Social work, Griffith University, Gold Coast, AU-QLD, Australia; 3 Cities Research Institute, Griffith University, Gold Coast, AU-QLD, Australia; 4 School of Information and Communication Technology, Griffith University, Gold Coast, AU-QLD, Australia; 5 Department of Health Economics, Wellbeing, and Society, Australian National University, Brisbane, AU-QLD, Australia; 6 School of Public Health, University of Queensland, Brisbane, AU-QLD, Australia

**Keywords:** climate, extreme heat, early warnings, older persons, Australia

## Abstract

**Background:**

Heatwave warnings provide crucial information about the nature of the event and the steps that can be taken to mitigate its impact. It is well known that heat events disproportionately impact the health of older adults. Therefore, it’s critical that heatwave warnings reach this population. However, our current understanding of the effectiveness of heatwave warning messages among older Queenslanders is limited.

**Methods:**

A Queensland wide survey was conducted in 2022 among 547 older adults (≥65 years), aiming to collect information on their perception of heat-related health risk, their knowledge of the existing heatwave warnings, and if they had ever heard of a heatwave warning. Chi-square analysis followed by multinomial or binomial logistic regression was utilized to understand various socio-economic and personal factors that impact the heatwave warning reach to older Queenslanders.

**Results:**

Only 43% of the respondents had heard a heatwave warning and only 49% of those who heard a warning(s) changed their behavior as a result. The results showed 20% of respondents perceived themselves to be at heat-related health risk, and these individuals were 1.98 times more likely to have heard heatwave warnings. Further, individuals who perceived themselves to be at heat-related health risk were 3.62 times more likely to adopt adaptive measures in response to heatwave warnings.

**Implications:**

This study suggests that in older adults, higher knowledge and perception of heat-related health risk are associated with higher likelihoods of attention to heatwave warnings and adoption of cooling measures.

## Introduction

Heatwaves, often defined as prolonged days of extreme heat, are projected to increase in intensity and frequency in the context of climate change, presenting a serious threat to the health and well-being of people around the globe.^1^ For a country like Australia with varied climate zones from tropical and sub-tropical to arid, if global warming continues at the current rate, it is projected that heatwaves will become 85% more frequent.^2^ Heatwaves in Australia are defined as three or more days of high maximum and minimum temperatures that are unusual for that location.^3^ Of all natural hazards impacting Australians, heatwaves are the most fatal, resulting in increased mortality and morbidity.^4-6^ Demand for health services such as hospital admissions and emergency visits, all increase during heatwave events,^7^ and a recent meta-analysis has shown an increase in the likelihood of ambulance callouts in Australia on heatwave days.^8^

The health impacts of heatwaves are often more pronounced within vulnerable populations, especially older people.^9^ Chronic conditions that are more prevalent in this population, such as cardiovascular, respiratory, neurological, and renal diseases along with reduced thermoregulatory response, put older adults at increased risk during heatwave days.^10,11^ These risks can be further exacerbated if the older adult belongs to a low socio-economic group or is experiencing social isolation or loneliness.^12^ Several recent Australian studies highlight the serious risks posed to older adults by heatwaves. A report from the Australian Institute of Health and Welfare (AIHW)^13^ summarizing national data on extreme weather events noted that of the 2150 hospitalizations attributed to extreme heat in Australia between 2019 and 2022, 37% were people over the age of 65 years. Another study found heatwaves were associated with an average national increase in mortality of 28% for people aged 75 or above in Australia, a rate that increased to 49 % in Brisbane, the capital city of Queensland.^14^

As our understanding of the devastating impacts of heatwaves has increased, there has been rising focus on the implementation of early warning measures to ensure people are better prepared. For example, in 2014 the Bureau of Meteorology (BoM) began issuing heatwave warnings and by 2015, the Queensland Government adapted heatwave warnings as part of their heatwave action plan. Early warning systems (EWS) are crucial and can play a key role in preventing the adverse effects of the extreme weather events such as heatwaves.^15,16^ Heatwave warnings constitute a crucial element within the framework of heatwave risk communication and heat EWS.^17^ Their primary objective is to augment the adoption of self-protective behaviors and elevate the precision of risk-related decisions, ultimately reducing heatwave associated mortality and morbidity.^17^ Previous studies have shown the implementation of heatwave warnings reduced the impact of heatwaves on all-cause mortality and morbidity.^18-21^ Evaluating the effectiveness of these warnings depends on the extent to which they are received, comprehended, and perceived as relevant by the public. Another key aspect in this evaluation is comprehensive understanding of the public’s responsiveness to heatwave warnings for optimizing the impact of such risk communication strategies and fortifying community resilience.^17^ Such understanding depends on factors such as individual concerns, lived experiences, and socio-economic factors.

The Australian state of Queensland is one of the most at-risk regions in the world for high-impact heatwaves.^22^ However, to date, there has been little research carried out in Queensland investigating if these early warnings have been heard by individuals, especially older people. Moreover, there is currently a lack of research examining whether heatwave warnings are associated with meaningful behavior change among those populations most at risk from heatwaves. Addressing this gap is particularly important as emerging evidence suggests that in Australia, heatwave communications—unlike public messaging about the risks of other natural disasters such as bushfires or floods, is commonly portrayed by media with pleasurable summer activities.^23^ Of concern is whether this positive association is leading to complacency regarding the health risks of heatwave events by minimizing a person’s own inherent perception of heat impacting their own health. This conscious change may be compounded further in older populations where the body’s unconscious thermal perception of hot ambient conditions diminishes as we age impacting behavioral responses.^24,25^

To begin addressing this research gap, as part of a larger project we surveyed 547 older Queenslanders to improve our understanding about older persons heat risk knowledge, heat risk perception, heat communication and warnings, technology use and cooling behaviors. This study focused on a subset of the larger survey. It first aims to improve our knowledge about whether heatwave warnings are reaching this large at-risk population. Second, our analysis of the survey data aims to better understand whether older Queenslanders who do receive heatwave warnings subsequently respond and take steps to change their behavior to mitigate heat health risks. The study also explored various factors impacting the reach and response to heatwave warnings.

## Methods

### Survey Design

**Figure 1.**
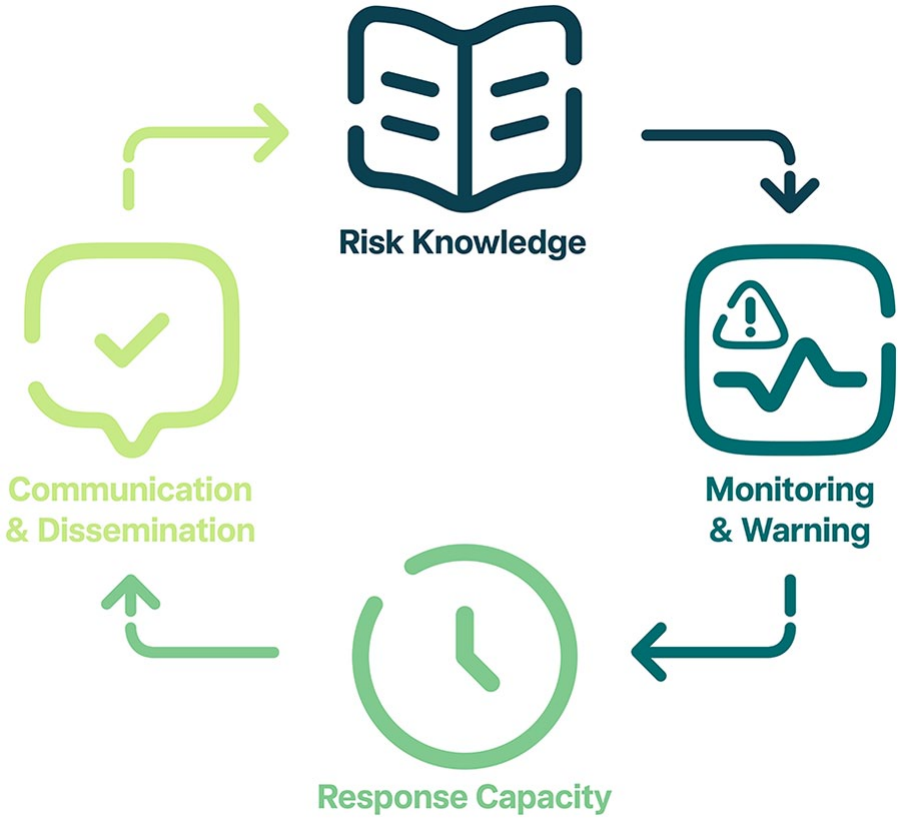
Garcia’s and Fernley’s EWS framework.^26^

The construct of the survey questionnaire drew on Garcia’s and Fernley’s^26^ early warning systems (EWS) framework (Figure 1). EWS are used in the disaster risk reduction field to provide timely warnings to populations at risk.^16^ These warning systems aspire to be people centered and include four key components: a) risk knowledge, ii) detection, monitoring, and forecasting, iii) building response capacity and preparedness, and iv) communication or dissemination of information. Our survey questionnaire is an omnibus survey designed with no specific hypothesis, but rather for collection of data on these four key areas including demographics. The sections were designed with three key objectives to: it) ascertain knowledge and attitude toward heat as a health problem, ii) identify attitudes toward and use of monitoring technologies, and iii) describe behaviors used when responding to heat stress and related messaging. These four categories were considered important to provide evidence of feasibility of an individualized heat-health early warning system for older persons as part of the larger project.^27^

A 144-question survey questionnaire was developed after a review of the literature on surveys conducted to gather information on heat wave risk, knowledge, and perception and digital literacy of older persons. Questions and response options were informed by previous surveys.^28^ All questions utilized elsewhere were adapted to the context of heat waves in Australia and designed for conduct with older persons, with a mix of both closed and open-ended questions.^28^ The draft questionnaire was cross-checked for content by discipline experts from statistics, heat-health epidemiology, thermophysiology, public health, information technology, and researchers with expertise in different aspects of the survey. This was followed by piloting of the survey among a selection of 43 (online version) and 10 (paper version) older Queenslanders for testing cognitive understanding of the content within the target population. Multiple iterations were tested, and revisions were made to the draft questionnaire to facilitate ease and understanding and to ensure survey content validity. The survey development did not involve any sensitivity or specificity statistical testing for checking the reliability of the omnibus survey.

### Study Location and Participants

The target population was people aged 65 years or above living in Queensland, the third most populous Australian state. The Queensland population is approximately 5.2 million, with older people accounting for 17% of this population.^29^ Assuming the population of older Queenslanders to be approximately 850 000, a survey sample of at least 384 was required for the results to meet a 95% confidence level with a 5% margin error (assuming 50% population proportion). Given that the survey was an omnibus survey without a specific hypothesis to test, the sample size was calculated based on above assumptions.

**Figure 2.**
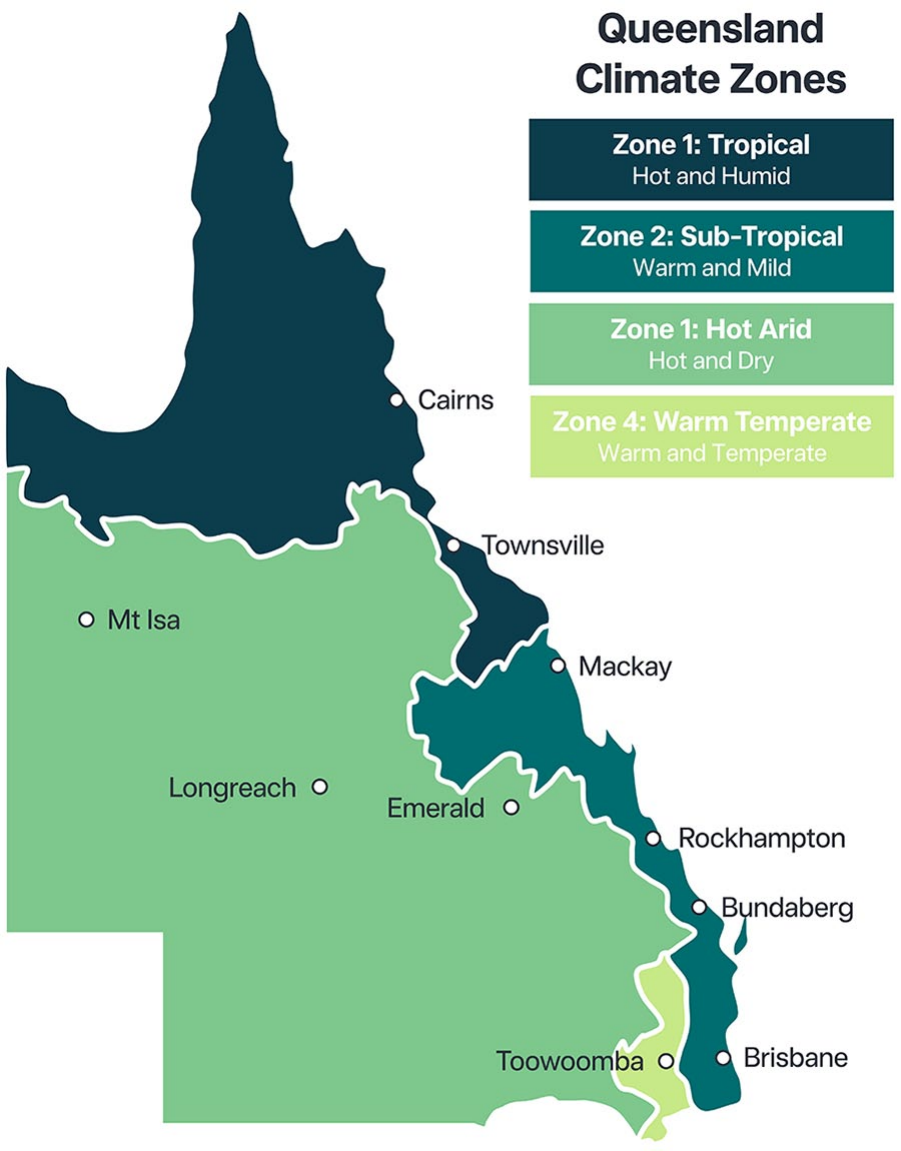
Climate zones in Queensland. Source: Queensland Government Department of Public works.^30^

Of the eight Australian climate zones, the Queensland state includes four climate zones ranging from tropical to hot arid with variations in temperature and humidity^30^ (Figure 2). These different climates mean that heatwaves can be experienced differently (ie, intensity, frequency, and duration), and people may use different strategies to cope.

To ensure adequate representation from these diverse climate zones, we applied a climate zone quota to our online sample. This was based on the proportion of population in each zone: 11% for climate zone 1, 81% for zone 2, 3% for zone 3, and 5% for zone 5.^31^

These quotas were applied on the calculated sample size (ie, n = 384). Age or gender quotas were not applied as our survey panel partner could not guarantee sufficient numbers of over 65-yearsalongside the prioritized climate quotas.

### Data Collection: Recruitment, Eligibility, and Administration

Participants were recruited utilizing both stratified sampling (proportional to climate zone) and convenience sampling method through two pathways: an online panel (climate zone), and through local media, newsletters, flyers in public places, and snowball methods across researcher networks. We included a paper mode for people aged 75 years or over. This age criteria was applied to the paper survey group based on findings of a national survey of information technology use in older people that indicated that people in this age group were more likely to have limited access to the internet and we wanted to ensure their sufficient representation in our survey.^32^ Online surveys were administered through Qualtrics (Qualtrics, Provo, UT).

For the paper survey, questionnaires were posted to recruited participants (who self-reported that they were aged 75 or over) together with the study information sheet. Participants were requested to return their completed questionnaire using a supplied replied-paid envelope and this constituted consent. No follow-up or reminder calls were made to the participants. For the online survey, participants were compensated by Qualtrics (Qualtrics, Provo, UT) and paper-based survey participants became eligible to enter a prize draw to win one of five AUD100 gift vouchers.

The research questions addressed in this paper are informed by a subset of the data collected as part of the statewide survey of older Queenslanders to explore their heat health risk perception, knowledge, adaptive/cooling behaviors, and digital literacy. The questions that inform the analysis reported in this paper are:

1. Are heatwave warnings reaching older persons in Queensland?
2. What factors impact the heatwave warnings being heard and acted on (what actions do older persons in Queensland take after hearing a heatwave warning)?

Answering these questions is key to providing guidance for improving communication around heatwave warnings in Queensland, which forms a major component of any early warning system.

### Data Analysis

The online and paper-based data was merged for data analysis. This merging was considered appropriate because the survey questionnaire was identical for both modes of survey administration and data collection. Moreover, few questions (K2 and K4) (see the questionnaire attached in the supplementary file) from the digital section were utilized to find if there were key differences in characteristics of paper and online respondents before merging the datasets.

### Quantitative

The data analysis was carried out in SPSS (28.0), where descriptive and bi-variate analysis were performed initially on the merged dataset. In bi-variate analysis, Chi square or Fisher’s exact tests (expected cell frequencies less than or equal to five) were used to test for the inter-relationships among variables with a *P*-value < .05 considered to be statistically significant. Initially, the descriptive (frequency) analysis of the heatwave warning related questions (section H: see questionnaire attached in the Supplementary File) was carried out followed by a basic chi-square analysis to explore if hearing a heatwave warning and behaving differently after hearing a heatwave warning (dependent variables) is related to:

1. Socio-demographic factors such as age, gender, education, income, self-reported health status, or living alone, and
2. Heat health risk perception, knowledge, or heat health experiences.

The variables which were found to be significantly statistically associated were then fitted into a multinomial and/or binomial logistic regression analysis based on the outcome/dependent variables (categorical variables) to further understand how various factors can impact heatwave warnings being heard and being acted upon. Before considering factors for this step of quantitative analysis (nominal (bi and or multi) regression analysis), all independent factors which were associated with outcome variables were further assessed to determine how closely they were associated with each other.

Assessing the associations between independent factors ensures the validity and reliability of regression analysis by detecting multicollinearity, aiding in model specification, facilitating interpretation of results, and guiding variable selection. If two independent variables were found to be closely associated with each other, we opted to prioritize one variable over the other based on theoretical relevance or empirical evidence, excluding the redundant variable from the analysis. Ultimately, the goal was to ensure that the selected independent variables were sufficiently independent to provide meaningful insights into the relationship with the outcome variable without compromising the integrity of the regression model.

The following questions were used an indicator/measure of heat health risk knowledge: E1.B- “*People suffering from chronic diseases (e.g., lung or heart disease are hospitalised less often when there are heatwaves*,” E5- “ Overall, *how much do you feel you know about the consequences of heatwaves on your health?*” Questions chosen for measure of heat health risk perception Included F1.7- “How *concerned are you about the heatwave affecting you or your family*,” F2- “*how serious a problem do you think heatwaves and extremely hot weather are for Australia?*,” and F7- “*Do you feel more at risk of heat than people of similar age to you?*” However, for carrying out the regression analysis F7 was used as an indicator of personal heat health risk perception.

Multinomial Logistic regression: To analyze the influence of personal heat health risk perception (independent variable with yes and no as categories) on heat warnings being heard, we employed a multinomial logistic regression model because the outcome variable was a categorical variable (3 categories: yes, no, and don’t know). The model was estimated using maximum likelihood estimation (MLE). We evaluated model fit through likelihood-ratio tests and examined the goodness-of-fit using Pearson and deviance statistics. Odds ratios (OR) were calculated to quantify the strength and direction of the association between the variables.

Binomial Logistic regression: *To analyze the influence of personal heat health risk perception (independent variable with yes and no as two categories) on heat warnings being acted on, we employed a binomial logistic regression model because the outcome variable was a binary variable (yes and no). The model was estimated using maximum likelihood estimation (MLE). We assessed model fit with the Akaike Information Criterion (AIC) and the Hosmer-Lemeshow test. Odds ratios (OR) were calculated to quantify the strength and direction of the association between the variables.*

For both multinomial and binary logistic regression models, “no,” was used as a reference category

### Qualitative

Data were collected through open ended responses for question H4 (If you behaved differently because of heatwave warning, please tell us what did you do differently?). Qualitative analysis was guided by Braun and Clarke’s framework for reflexive thematic analysis.^33^

In the first phase, two researchers read through all the responses multiple times to familiarize themselves with the data. In phase two, initial coding was completed by one researcher (MO). An inductive approach was adopted at this stage and the focus was on producing brief and succinct codes that clearly spoke to the respondents’ behaviors after hearing heatwave warnings. Once this initial coding phase was completed, two researchers (MO and SR) commenced phase three analysis by separately grouping the codes into themes and sub-themes that helped to illuminate various aspects of the respondents’ experiences of heatwave warnings. Themes tended to coalesce around the various adaptive actions (from individual to household) reported by the older adults completing the survey and the decision was made at this point to begin counting the frequency of key terms related to an adaptive measure (eg, key words such as water or hydration being mentioned in relation to the “Personal Cooling” theme). It was felt these keywords could help to further inform the thematic analysis by providing an indication of the frequency of keywords relating to each the themes. Any differences arising in the coded themes were addressed by the two researchers through discussion and this served as an initial stage in phase four of the analysis (reviewing potential themes). Leximancer was used as a tool to assist with concept mapping of the themes at this stage of the analysis and both themes and sub-themes were grouped by both researchers until a final set of named and defined themes were completed (phase five). At this point, work began on writing the paper (phase six). As the ability to report qualitative findings was limited within the context of the full survey results, key quotes that are representative of the themes were selected to illustrate the broader themes and a table was created to allow keywords and keyword frequency to be added to the final list of themes.

## Results

### Participation Rate

Combined online and paper-based survey responses resulted in a sample size of 547. For the online survey a total of 447 responses were received, with complete responses for 412 (92% [412/447] completion rate). For the paper survey, 201 surveys were posted to participants who had expressed an interest in the project, 138 (69% [138/201] paper-survey response rate) were received of which 135 were complete (98% [135/138] completion rate).

### Socio-Demographic Characteristics

The 547 older adults can be considered as representative sample of older adults in Queensland based on comparison with existing population-based data for (age group, self-reported health and finance status, and gender ratio) older Queenslanders. Although we made effort to ensure it was a representative sample of the Queensland population, the education attainment status of our sample was slightly different from that of the total Queensland population. Table 1 shows the socio-demographic representation of the sample as compared to the Queensland population. Recruiting people aged 75 or over through paper mode, to make sure that our survey reached this population resulted in over representation of age group 75 to 79 in our survey. More males (53%) completed the survey as compared to females (47%). Similar to the Queensland population,^29^ almost a third of respondents were born-overseas with places of birth ranging from the United Kingdom (50%), New Zealand (13%), South Africa (1%), India (1%), China (1%), and 34% reporting their country of birth as “other.” These others included several countries from USA, Germany, Hungary, France, Netherlands, Egypt, Sri Lanka, Thailand, Belgium, and Romania. Data relating to self-reported health status showed that 38% of the study sample reported they have good health as compared to 41% in a large and recent Australian National Climate Action survey (CAS)^34^; 87% of the sample self-reported having at least one chronic health condition as compared to 80% reported by the Australian Institute for Health and Welfare.^13^

**Table 1.** Comparative Analysis of Study Sample and Wider Queensland (QLD) Population Demographics and Education Levels.^13,29,34^

Variable	% Study sample		% QLD population	
Age group				
65-69	24		30	
70-74	29		28	
75-79	28		19	
80-84	12		12	
85+	7		11	
Self-reported financial status				
Struggling financially	17		17	
Doing okay	47		47	
Comfortable	33		30	
Financially well off	3		6	
CALD representation	30		29	
Gender				
Male to female ratio	53:47		49:51	
Education level	65-74	75+	65-74	75+
Bachelor and above	24	27	15	9
Certificate III, IV, diploma	38	33	27	22
Year 10 and above or certificate I/II	36	35	39	36
Year 9 and below secondary	2	5	19	33

Self-reported financial status in the sample was comparable to the Queensland population. Table 1 also depicts the representativeness of the sample in terms of education level. The study sample was more educated when compared to the Australian Bureau of Statistics (ABS) population data, however, this is not an uncommon finding for survey samples.^35,36^ Most survey respondents (85%) were retired, and 31.6% lived alone. Furthermore, 22% reported having one or more disabilities, with 28% requiring assistance with household tasks; 24% of surveyed participants reported feeling lonely and isolated based on the UCLA Loneliness Scale^37^; 20% of respondents resided in rented houses and 16% lacked air conditioning.

### Heatwave Warnings Reach and Response

The survey results showed that 47% of our sample had not heard a heatwave warning and a further 10% were unsure if they had heard a warning or not. Moreover, even among the 43% (236) of respondents who *had* heard a heatwave warning, only 49% (115/236) behaved differently as a result of hearing the warning, which forms only 21% (115/547) of the total sample.

**Figure 3.**
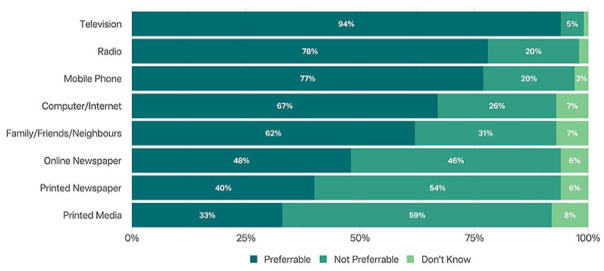
Preference for media channel to get heatwave related information.

As shown in Figure 3, 94% of the survey respondents reported they would prefer television for receiving heatwave warnings or heat preparedness information, followed by radio (78%) and mobile phones (77%), respectively. In alignment with their preferences, television (40%) was the most common source from which the respondents were currently obtaining heat-related information or heatwave warnings, followed by radio (17%).

Factors associated with the reach of heatwave warnings: Chi-square analysis revealed a statistically significant association between the awareness of heatwave warnings and respondents’ knowledge and perceptions regarding heatwaves. Additionally, experiences related to heatwaves during past summers emerged as influential factors in determining the likelihood of hearing these warnings (Table 2). Conversely, socio-demographic factors, including age, gender, education, self-reported financial and health status, and living arrangements, exhibited no statistically significant relationship to the reception of heatwave warnings (*P* > .05).

**Table 2.** Chi square analysis results (chi square value (*P* value)) showing significant relationship between hearing heatwave warnings and heatwave experiences, knowledge, and perception (concern). Alpha (α) set to 0.05.

Hearing heatwaves	Experienced heatwaves in past 12 months	Knowledge of heatwave consequences	Concern for region Concern for Australia where they live	Personal concern
	14.838 (*P* = .022)	32.980 (*P* < .001)	14.355 (*P* = .006) 16.383 (*P* = .037)	9.322 (*P* = .009)

Further exploration through Chi-square analysis highlighted substantial statistically significant relationships (*P* < .05) between previous heatwave experiences, heatwave knowledge, and perception, and behavior change following a heatwave warning. Willingness to change behavior as a result of receiving heatwave warnings also demonstrated a statistically significant association with self-reported health (8.55, *P* = .036) and financial status (8.627, *P* = .035).

To investigate further, multinomial and/or binary regression models were employed to examine the relationships between the heat risk perception (independent variable) and dependent variables (hearing heatwave warnings [H1] [multinomial model] and changing behavior after hearing heatwave warnings [H3] [binary model]). Multinomial analysis revealed that respondents perceiving themselves at risk were found to be 1.98 (95% CI: 1.26-3.12) times more likely to hear heatwave warnings when compared to those who do not perceive themselves at risk. Moreover, this increased awareness translated into a substantial 3.62-fold (95% CI: 1.91-6.84)—increase in the likelihood of adopting cooling behaviors in response to these warnings, as revealed through binomial regression analysis.

To minimize the impact of multicollinearity on our study outcomes, we intentionally excluded certain related variables, such as knowledge and experience, from the regression models. This strategic omission was implemented to ensure the robustness and independence of the selected predictors, thereby enhancing the reliability and interpretability of the obtained results.

### Heat Health Risk Knowledge, Perception, and Experience in Older Persons in Queensland

Chi-square analysis revealed a statistically significant association (*P* < .05) between the reception of heatwave warnings and factors such as heat-health risk knowledge, perception, and previous experiences among the surveyed cohort. However, heat health risk knowledge within the respondent group was notably low, with 75% of participants lacking awareness of the potential consequences of heatwaves on their health.

Furthermore, a substantial portion of the sample (87%) self-reported having one or more chronic health conditions, with 70% indicating chronic health issues, including cardiovascular, respiratory, renal, or mental health concerns known to be exacerbated by extreme heat.^11,38^ Despite the high prevalence of chronic health conditions, 30% of respondents were unaware that having a chronic health condition increased their likelihood of hospitalization and heightened their vulnerability to heatwaves. Additionally, 10% did not acknowledge this correlation.

In terms of perception about heatwaves, a majority of respondents (80%) perceived themselves to be at lower risk compared to others of their age group. Notably, their concern about the impacts of heatwaves on a broader scale, such as on Australia as a whole (35%), was greater than the concern about the effects of heatwaves on themselves or their families (18%). This observation is particularly interesting given that a substantial proportion (79%) reported experiencing heat-related health symptoms in the past.

### Cooling Actions After Hearing a Heatwave Warning

Based on the qualitative analysis, in response to the heatwave warnings, respondents exhibited adaptive behavior that can be represented by four themes with associated keywords (Table 3). Commonly embraced strategies encompassed: ensuring adequate hydration, seeking refuge indoors, diminishing physical exertion, and employing cooling devices—such as fans and air conditioners—to maintain a cool indoor environment.

**Table 3.** Analysis of Responses Indicating Key Themes in Heatwave Coping Strategies.

Responses coded	Keyword frequency	Themes	Statements supporting the themes
Hydration (sometimes temperature of water mentioned)	48	Personal cooling	*“I ensured that I . . . had plenty of water on hand so as not to dehydrate. . .”* *“Ensured had plenty of cold water in the fridge”*
Stay indoors	34	General protective behaviors	*“made it a quiet day at home and stayed indoors”* *“Closed all curtains/blinds and stayed indoors”*
Used cooling (including unspecified method)	29	Active and personal cooling	*“installed more fans”* *“put face washers in the freezer and put them on my face to cool me down”*
Reduce activity	22	General protective behaviors	*“. . . abandon any unnecessary physical activity, drink regularly and use the AC or sit in a breeze.”* *“try to do nothing on the day when the heatwave comes”*
Stay indoors with air conditioning	20	Active cooling	*“. . . instead of doing gardening as planned I stayed indoors with the aircon on.”* *“. . . I used fans or air-conditioning if necessary”*
Changed (or considered changing) plans to reduce heat exposure	13	General protective behaviors	*“I rearrange my plans so that I am not out in the heat longer than I have to be.”* *“Did not go for my usual ½ hour walk”*
Changed clothing, including wearing a hat	11	Personal and passive cooling	*“I opened up the house before the heatwave to cool it down as much as possible and closed the house up during the heatwave to stop the heat entering. I . . . wore loose, light-weight clothing.”* *“Just dressed in cooler clothes and drank more water than usual”*

## Discussion

The study found a significant gap in the awareness and response to heatwave warnings among the respondents. Nearly half of the sample had not heard a heatwave warning, and only 21% of the total sample took action after hearing one. Television emerged as the preferred medium for receiving heat-related information, although many relied on radio and mobile phones. The regression analysis revealed that awareness and behavioral responses to heatwave warnings were strongly associated with individuals’ knowledge, perceptions, and past experiences with heatwaves. In contrast, socio-demographic factors like age, gender, and education did not significantly influence the reception of warnings.

The survey results clearly indicate that the awareness of ongoing heatwave warnings among older Queenslanders was relatively limited, highlighting the fact that at the time of survey these warnings were not reaching this vulnerable population and pointing to the need for enhanced communication strategies. It is important to consider the impact of the concurrent La Nina effect in the Southern Hemisphere during survey implementation.^39,40^ This climatic variation can potentially contribute to limited heatwave like conditions due to cooler summers accompanied with heavy rainfall^39,40^ which may lead to diminished perception of the heatwave and related risks, emphasizing the importance of tailored and sustained communication efforts to ensure the safety of older individuals during seasonal extreme weather events.

Similar to the findings of previous national and international research,^21,41-44^ that highlight the importance of heat health risk perception in determining the effectiveness of heatwave warnings, findings of the survey underline the critical role of perceived heat health risk in shaping both the reception and subsequent actions following heatwave warnings. Our study reveals that the perceived risk and heat health knowledge was low among the survey respondents. Three quarters of the sample was unaware of the consequences that heatwaves can have on their health. The incidence of self-reported heat exacerbated chronic health conditions in the sample was very high however, one third of the respondents did not know this increases their risk of being hospitalized as heat can exacerbate those conditions. More than three-quarters of respondents self-reported experiencing heat related health symptoms during the previous summer seasons, but still did not perceive themselves to be at risk of heat-health impacts. This highlights the lack of knowledge around chronic health conditions and heat vulnerability, which in turn identifies the need 1) to create awareness regarding heat as a health issue and 2) the need for primary healthcare providers to talk with this vulnerable group about how extreme weather events as heatwaves can be detrimental to their health as called for in the National health and Climate Strategy.^45^

Most respondents (80%) did not perceive themselves to be at risk due to heat but did see other people their age being at risk, highlighting the phenomenon of cognitive dissonance. This is consistent with findings of,^46-49^ that highlight that older people do not see themselves at risk of heat or heat being a threat to their health, instead heat and its related health risks are seen as a threat for their peers. Additionally, respondents considered heatwaves to be more a concern for Australia overall, rather than for them personally or their family. This lack of heat health risk perception can be attributed to multiple factors. Firstly, “heat” is often viewed as a positive and normal phenomenon which shapes Australian cultural identity and is a characteristic of the typical Australian summer encompassing holidays, sport, beaches, and ice-cream.^23^ Secondly, when it comes to public messaging in comparison to other natural disasters, such as cyclones, floods, or bushfires, which are more destructive and less frequent, heatwave risks are often normalized, and they are considered as part of life.^23^ Thirdly, the lived experience of typically hot summers and no ill effects throughout a person’s childhood and healthy adulthood contrasts with inevitable age-related decrements in physiological capacity to deal with heat, largely determining an individual’s risk.^50^

Furthermore, our respondents reported that they rely on mainstream media (ie, television) for information, as a preferred source of heatwave warnings or heat related information, over other sources like mobile phones. Therefore, an additional factor which might further contribute to lower risk perception and knowledge is the positive imagery consistently employed by the mainstream media to portray summers and high temperatures. This is evident in the outcomes of an exploratory analysis of Australian TV news coverage, where heatwave warnings coincide with positive imagery featuring beaches and waterways, conveying a narrative of low-risk outdoor activity. Notably, this visual representation often overshadows critical information on health warnings, narratives, and guidance, along with the potential adverse health consequences.^23,51-53^ Research has suggested that media strategies employing negative imagery and visuals of heat to highlight the threat posed by this silent killer^47^ can play an important role in impacting the heat risk perception especially, in older persons, many of whom still rely on mainstream media for information.

Heat-health warning systems are operating in many countries to mitigate the impact of heatwaves on human health,^54^ but to increase the effectiveness and reach of these systems, warnings need to be tailored and customized, especially for vulnerable groups such as older adults. Our survey results show that people who are hearing these warnings are taking preventive measures to be heat safe. Therefore, exposure of particular groups to tailored messages could result in improved reception of the heatwave warnings. Along with the general population based early warning systems in place, there is increasing call^55^ to better individualize early warning systems which take into consideration individual factors such as individual exposure, vulnerability, and sensitivity to heat, rather than relying entirely on population level warnings.

Moreover, there is a need to increase perception of heat and its related health risks in the population so as to optimize reception of warnings and corresponding actions. Investing in targeted communication and community awareness campaigns along with working with the media stories focusing on heatwaves and related health risks is a key component of effective early warning systems to better balance negative and positive heatwave imagery.

The significant findings from this statewide survey, should be considered in light of key study limitations. Despite extensive piloting for content and construct validity, one limitation is the use of an untested and unvalidated questionnaire to collect information on these heat early warning topics from the sample, hence survey reliability construct could not be assessed. Another limitation of note is the self-selection of the participants for the paper-based survey potentially resulting in positive response bias. Moreover, as the study was cross-sectional, the findings did not imply causality and similar to other survey studies, the information collected was self-reported, which could have been subject to information bias. An additional uncontrolled factor, which might have impacted survey results were the La-Nina conditions at the time of the survey (and the 3 years preceding it)^56^ which may have introduced recall bias around heat awareness and experience. Another point to add is that the sample was not representative of population in terms of education.

## Conclusion

In conclusion, heatwave warnings form a crucial component of heatwave risk communication and early warning systems, aiming to bolster the adoption of self-protective behaviors and enhance the precision of risk-related decisions. However, the effectiveness of these warnings relies heavily on their reception and perceived relevance by the public. Findings of this survey highlight the significant influence of individual perceptions of heat-related health risks on the efficacy of heatwave warnings. While lived experiences and socioeconomic variables undoubtedly contribute to this efficacy, the pivotal role of self-perceived risk cannot be overstated. Notably, individuals’ perception of their own susceptibility to heat-related health issues can supersede the influence of these broader contextual factors. Consequently, efforts to enhance awareness of heat as a health risk could prove instrumental in bolstering individuals’ perception of their susceptibility, thereby amplifying the effectiveness of heatwave warnings.

The implications of these findings accentuate the urgent need for targeted public health and policy interventions. Addressing population gaps in heat health risk knowledge, dispelling misconceptions about risk, and aligning individual risk perceptions with evidence-based health impacts are crucial steps. Tailored efforts to both improving population heat risk awareness and enhancing the effectiveness of heatwave warnings among older populations are imperative for fostering adaptive behaviors and ultimately improving heatwave communication. These interventions are not only essential for the well-being of older individuals but also contribute to overall community resilience in the face of increasing heat-related challenges.

## Supplemental Material

sj-pdf-1-jpc-10.1177_21501319241286584 – Supplemental material for Towards Improvement of Heatwave Warnings for Older Adults: The Case of Queensland Australia Supplemental material, sj-pdf-1-jpc-10.1177_21501319241286584 for Towards Improvement of Heatwave Warnings for Older Adults: The Case of Queensland Australia by Mehak Oberai, Steven Baker, Aaron J. E. Bach, Connor Forbes, Ella Jackman, Sebastian Binnewies, Zhiwei Xu, Sarah Cunningham, Son Nghiem, Dung Phung and Shannon Rutherford in Journal of Primary Care & Community Health
