# Supplementary material for: Towards Improvement of Heatwave Warnings for Older Adults: The Case of Queensland Australia
Source: J Prim Care Community Health. 2024 Nov 4;15:21501319241286584. doi: 10.1177/21501319241286584 (PMC11536501; doi:10.1177/21501319241286584)
Supplement: sj-pdf-1-jpc-10.1177_21501319241286584 – Supplemental material for Towards Improvement of Heatwave Warnings for Older Adults: The Case of Queensland Australia [file sj-pdf-1-jpc-10.1177_21501319241286584.pdf]

COMPLETION AND MAILING OF THE QUESTIONNAIRE WILL BE TAKEN AS YOUR INFORMED CONSENT TO PARTICIPATE IN THIS STUDY.

Date of completion: ____/____/2022 Start time: ________

Please read all questions carefully because no two questions are identical. Sometimes two questions may seem similar, but this is essential for reliability purposes.

Section I

It will be good to know about you to begin with, let's start ☺

A1 Gender (Please select the correct option)

- ☐ Male
- ☐ Female
- ☐ Other
- ☐ Prefer not to say

A2 In which age category do you fit (Please select the correct option)

- ☐ 65-69 yrs.
- ☐ 70-74 yrs.
- ☐ 75-79 yrs.
- ☐ 80-84 yrs.
- ☐ 85 and above

A3 Your postcode ____________________

A4 Were you born in Australia?

- ☐ Yes, go to A5
- ☐ No, go to A7

A5 In which Australian state or territory were you born?

- ☐ NSW
- ☐ WA
- ☐ SA
- ☐ ACT
- ☐ QLD
- ☐ NT
- ☐ VIC
- ☐ TAS

A6 Do you identify as:

- ☐ Neither Aboriginal nor Torres Strait Islander
- ☐ Aboriginal
- ☐ Torres Strait Islander
- ☐ Both Aboriginal and Torres Strait Islander

A7In which country were you born? (skip if born in Australia)

- ☐ New Zealand
- ☐ Indonesia
- ☐ United Kingdom
- ☐ India
- ☐ China
- ☐ South Africa
- ☐ Brazil
- ☐ Other, please specify__________________

B1 Which language(s) do you usually speak at home? (Please select all that apply)

- ☐ English
- ☐ Mandarin
- ☐ Italian
- ☐ Cantonese
- ☐ Vietnamese
- ☐ An Aboriginal language, please specify__________________
- ☐ A Torres Strait Islander Language, please specify__________________
- ☐ Other, please specify__________________

B2 What is your highest education level?

- ☐ Postgraduate diploma/ certificate/degree
- ☐ Bachelor's degree

☐ Diploma or Advanced Diploma
☐ Certificate Level III or IV
☐ Secondary school education
☐ Primary school education
☐ None of the above

B3 Which of the following describes your living situation? Please select all that apply.

☐ Living alone
☐ Living with partner
☐ Living with other family members (e.g., parents, extended family, adult/ children)
☐ Living in a shared household
☐ Living in an aged care facility
☐ Other ___________________

C1 Which of the following best describes how you spend your time? (Please select all that apply)

☐ Working – full time (35+ hours per week)
☐ Working – part time
☐ Working on a casual basis
☐ Unemployed and seeking work
☐ Retired
☐ Unpaid work- looking after house/children/dependents
☐ Not in paid employment due to a disability
☐ Student- not in paid employment
☐ Other, please specify____

C2 What is your individual after tax income?

☐ Less than $10,399 per year

- ☐ $10,400-$15,599 per year
- ☐ $15,600-$20,799 per year
- ☐ $20,800-$31,199 per year
- ☐ $31,200-$41,599 per year
- ☐ $41,600-$51,999 per year
- ☐ $52,000-$64,999 per year
- ☐ $65,000-$77,999 per year
- ☐ $78,000 – $103,999 per year
- ☐ More than $104,000 per year

C3 How would you describe your current financial situation?

- ☐ I am struggling financially
- ☐ I am doing okay
- ☐ I am comfortable
- ☐ I am financially well off

C4 Is your home (Please select the appropriate option)

- ☐ A separate house
- ☐ A semi-detached house/townhouse
- ☐ Flat/apartment/unit
- ☐ Aged care facility (if this is selected go to D1)
- ☐ Other, please specify ____________________

C5 Do you live in (Please select the appropriate option)?

- ☐ Your own house/dwelling/home (go to C7)
- ☐ A rented house/dwelling/home (go to C6)
- ☐ Do not have a home/dwelling to live in (go to D1)
- ☐ Other, please specify ____________________

C6 If you rent, is it?

- ☐ Government rental (e.g., public housing)

☐ Private rental

C7 For how long you have been living in this dwelling/house?

☐ Less than 1 year

☐ 1-5 years

☐ 6-15 years

☐ More than 15 years

C8 What was your estimated electricity bill in the most recent quarter? (Please select the closest option)

☐ Less than equal to $160

☐ $161-$200

☐ $201-$240

☐ $241-$280

☐ $281- $320

☐ $321- $360

☐ $361- $400

☐ $401- $440

☐ More than $440

☐ Do not directly pay for electricity

☐ Do not know

D1How would you rate your health?

☐ Extremely Poor

☐ Poor

☐ Okay

☐ Good

☐ Very good

D2 Have you been diagnosed with any of the following chronic conditions? (Please select all that apply)

☐ Diabetes

☐ Heart problems (e.g., heart attack, stroke)

☐ High blood pressure

☐ Kidney or renal problems

- ☐ Respiratory problems (e.g., asthma, shortness of breath)
- ☐ Depression, anxiety, memory loss, or other mental health issues
- ☐ Alzheimer's disease
- ☐ Arthritis
- ☐ Back problems
- ☐ Any other condition that is not listed above, specify please__________________
- ☐ No, I do not suffer from any of the above

D3 Please tick the most appropriate option from hardly ever to often for the following questions:

	Hardly ever	Some of the time	Often
How often do you feel that you lack companionship?	☐	☐	☐
How often do you feel left out?	☐	☐	☐
How often do you feel isolated from others?	☐	☐	☐

D4 Do you need help with household tasks?

- ☐ Yes, go to D5
- ☐ No, go to D6

D5 Who do you normally ask for help? (Please select all that apply)

- ☐ Family member
- ☐ Friend
- ☐ Neighbour
- ☐ Paid Carer
- ☐ No-one

- ☐ Other, please specify ___________________

D6 Do you have a disability? (A disability includes sensory, intellectual, neuro-diverse, physical and mental illness – where the disability is permanent or is likely to be permanent).

- ☐ Yes, please specify ___________________
- ☐ No

D7 Do you use any of the following aids for getting around? (Please select all that apply)

- ☐ Walking aid
- ☐ Wheelchair
- ☐ Scooter/ Gopher
- ☐ No aids
- ☐ Other, please specify ___________________

Great work so far, 25% done, now we would like to hear about your views on heat and heatwaves. A heat wave or an extreme heat event is a period of unusually and uncomfortably hot weather. 😉

Section II

E1 Please answer to the best of your knowledge or belief for the statements below:

a. Health impacts from heat increase, if after a hot day, temperature remains high at night.

□ True □ False □Don't know

b. People suffering from chronic diseases (e.g., lung or heart diseases) are hospitalized less often when there are heat waves.

□ True □ False □Don't know

c. Heat can affect your health even before you feel any of the warning signs.

□ True □ False □Don't know

d. The increased temperatures associated with climate change are resulting in increased deaths and hospitalisations around the world.

□ True □ False □Don't know

e. Health impacts from heat increase following prolonged periods of very hot weather.

□ True □ False □Don't know

E2 When do heatwaves have the greatest effect on people's health? (Please select the most appropriate option to the best of your knowledge or belief)

□ In the beginning of the summer season

□ At the end of the summer season

□ All summer

□ Don't Know

E3 Some people are more sensitive to extreme heat. Select the groups of people who you think have a higher risk for health effects due to extreme heat? (Please select all that apply)

- ☐ Young adults
- ☐ People who work indoors
- ☐ People who perform a lot of physical activity (sports, construction workers)
- ☐ People aged 65 and above
- ☐ Very young children
- ☐ Pregnant women
- ☐ People who are socially isolated
- ☐ People who have a chronic disease

E4 Overall, how much do you feel you know about the causes of heatwaves?

- ☐ Nothing at all
- ☐ Virtually nothing
- ☐ A little
- ☐ Quite a lot
- ☐ A great amount

E5 Overall, how much do you feel you know about the consequences of heatwaves on your health?

- ☐ Nothing at all
- ☐ Virtually nothing
- ☐ A little
- ☐ Quite a lot
- ☐ A great amount

F1 How concerned are you that each of the following threats might directly affect you or your family? (Please tick the appropriate box for each of the following)

	Not At All Concerned	Not very concerned	Somewhat concerned	Fairly concerned	Very Concerned
Bushfires	☐	☐	☐	☐	☐
Cyclones	☐	☐	☐	☐	☐
Floods	☐	☐	☐	☐	☐
Crime	☐	☐	☐	☐	☐
Sea level rise	☐	☐	☐	☐	☐
Droughts/Water shortages	☐	☐	☐	☐	☐
Heatwaves	☐	☐	☐	☐	☐
War/International conflicts	☐	☐	☐	☐	☐
Food affordability	☐	☐	☐	☐	☐
COVID-19	☐	☐	☐	☐	☐
Impacts of climate change, generally	☐	☐	☐	☐	☐

F2 How serious a problem do you think heatwaves and extremely hot weather are for Australia?

- ☐ Not at all serious
- ☐ Somewhat serious
- ☐ Extremely serious

F3 On an extremely hot day, how would you rate the indoor temperatures of your home on the following scale?

- ☐ Cold
- ☐ Cool
- ☐ Slightly cool
- ☐ Neutral
- ☐ Slightly warm

- ☐ Warm
- ☐ Hot

F4 During the middle of a normal summer day, how would you rate the indoor temperatures of your home on the following scale?

- ☐ Cold
- ☐ Cool
- ☐ Slightly cool
- ☐ Neutral
- ☐ Slightly warm
- ☐ Warm
- ☐ Hot

F5 How vulnerable do you think the region within 50 km of your home is to the impacts of extreme hot weather?

- ☐ Not at all vulnerable
- ☐ Not very vulnerable
- ☐ Somewhat vulnerable
- ☐ Fairly vulnerable
- ☐ Highly vulnerable

F6 How sensitive are you to heat?

- ☐ Very sensitive
- ☐ Sensitive
- ☐ Not very sensitive
- ☐ Not sensitive at all

F7 Do you feel more at risk of heat than people of similar age to you?

- ☐ Yes, go to F8
- ☐ No, go to F9

F8 Please explain why you feel more at risk.

F9 Have you ever been told by a health professional that your health problems can make you more sensitive to heat?

- ☐ Yes
- ☐ No
- ☐ Don’t know
- ☐ Don’t have any health problems

G1 *Within the past twelve months*, have you been affected by extremely hot weather?

- ☐ Not affected at all
- ☐ A little affected
- ☐ Somewhat affected
- ☐ Badly affected

G2 *Prior to the past twelve months*, have you been affected by extremely hot weather?

- ☐ Not affected at all
- ☐ A little affected
- ☐ Somewhat affected
- ☐ Badly affected

G3 Have you ever experienced any of the following associated with hot weather (Please select all that apply)

- ☐ Anxiety
- ☐ Loss of balance/feeling dizzy/faint
- ☐ Headache
- ☐ Nausea/Vomiting
- ☐ Shortness of breath
- ☐ Irregular heart rate/rapid pulse
- ☐ Skin issues (e.g., prickly heat rash)
- ☐ Dehydration
- ☐ Muscle cramps
- ☐ Fatigue

- ☐ Changes in urination (e.g., Decreased frequency/ darker colour/smaller amount)
- ☐ Loss of appetite
- ☐ General weakness
- ☐ Lack of sleep/ Trouble sleeping/sleeping disturbance
- ☐ Other, please specify_________________
- ☐ Experienced none of the above

Well done 😊, you are halfway through. Relax a bit and have a stretch. Next, we would find out how you respond during hot weather.

Section III

H1 Have you ever heard a heatwave warning?

- ☐ Yes, go to H2
- ☐ No, go to H6
- ☐ Don't know, go to H6

H2 When did you hear about the heatwave warning?

- ☐ At least a night before the event
- ☐ Day of the event
- ☐ After the event
- ☐ Heard the warnings repeatedly
- ☐ Don't recall

H3 Did you behave differently because of the heatwave warning?

- ☐ Yes, got to H4
- ☐ No, go to H5

H4 Please tell us what you did differently?

H5 From which of the following sources did you obtain heat related information or heatwave warnings? (Tick all that apply)

- ☐ Television
- ☐ Radio
- ☐ Mobile phone
- ☐ Newspaper (printed)
- ☐ Newspaper (on computer)
- ☐ Internet/computer websites
- ☐ Social media
- ☐ Weather apps
- ☐ Printed material (posted or picked up)
- ☐ State Emergency Services (SES)
- ☐ Family/friends/neighbours
- ☐ Other, please specify__________________

H6 We are interested in your ideas about the ways heat warnings and heat preparedness information are provided. To what extent do you prefer each of the following sources for such information? (Please tick the appropriate box for each)

	Not preferable	Somewhat preferable	Very preferable	Don' t know
Television	☐	☐	☐	☐
Radio	☐	☐	☐	☐
Mobile phone	☐	☐	☐	☐
Newspaper (printed)	☐	☐	☐	☐
Newspaper (on computer)	☐	☐	☐	☐

Internet/ computer websites	☐	☐	☐	☐
Printed material (posted or picked up)	☐	☐	☐	☐
Family/friends/neighbours	☐	☐	☐	☐

I1 Do you feel confident in seeking help during extreme hot weather if you are not feeling well?

- ☐ Yes, go to I2
- ☐ No, go to I3

I2 Who would you contact for help if you are not feeling well due to hot weather (Please select all that apply)?

- ☐ Family
- ☐ Friends
- ☐ Neighbours
- ☐ Personal Carer
- ☐ GP or nurse
- ☐ Other, please specify___________________

I3 During extremely hot weather, how often do other people contact you to check on your well-being?

- ☐ Often
- ☐ Sometimes
- ☐ Most of the time
- ☐ Never

I4 Which of the following do you have at your home to reduce the effects of hot weather? (Please select all that apply)

- ☐ Air conditioning
- ☐ Fans
- ☐ Blinds and awnings
- ☐ Large windows and doors
- ☐ Large windows and doors with insect and/or security screens
- ☐ Outdoor living areas like Verandas/Decks/ Patios
- ☐ Ceiling insulation

- ☐ Wall insulation
- ☐ Roof overhang/ wide eaves
- ☐ Shady Plants
- ☐ Not any
- ☐ Other, please specify__________________

I5 Have you made changes to your home to make the temperature more comfortable during the hot weather?

- ☐ Yes, if yes go to I6 and skip I7
- ☐ No, go to I7

I6 What changes have you made, select from the options below (Please select all that apply)?

- ☐ Installed air conditioning
- ☐ Installed fans
- ☐ Purchased mobile coolers or pedestal fans
- ☐ Installed blinds and awnings
- ☐ Installed insect and or security screens to your windows/doors
- ☐ Added outdoor living areas like verandas/decks/ patios
- ☐ Installed ceiling insulation
- ☐ Tinted your windows
- ☐ Installed a light colour roof
- ☐ Added shady plants
- ☐ Other, please specify_____

I7 What is the reason you did not make any change? (Please select all that apply)

- ☐ Rental dwelling
- ☐ Could not afford it
- ☐ Didn't know how
- ☐ Not physically able
- ☐ Did not feel it was needed
- ☐ Other, please specify_______

I8 On a very hot day, how often do you use the following to maintain comfortable temperatures? (Please tick the appropriate box for each option)

	Never	Rarely	Sometimes	Frequently	Every time	N/A
Turn on the air conditioner	☐	☐	☐	☐	☐	☐
Turn on fans	☐	☐	☐	☐	☐	☐
Stay inside your house during the warmest times of the day	☐	☐	☐	☐	☐	☐
Keep windows closed when outdoor temperature is higher than indoor	☐	☐	☐	☐	☐	☐
Open doors and windows	☐	☐	☐	☐	☐	☐
Close blinds and curtains	☐	☐	☐	☐	☐	☐
Adjust your clothing (light materials, light colours, less clothing, loose clothing)	☐	☐	☐	☐	☐	☐
Increase intake of fluids (water/soft drinks)	☐	☐	☐	☐	☐	☐
Cool your body by taking showers or swimming	☐	☐	☐	☐	☐	☐
Use a wet cloth (on neck or face)	☐	☐	☐	☐	☐	☐
Reduce alcohol intake	☐	☐	☐	☐	☐	☐
Change the type of food I eat	☐	☐	☐	☐	☐	☐
Avoid physical activity	☐	☐	☐	☐	☐	☐
Avoid outdoors	☐	☐	☐	☐	☐	☐
Go outdoors at home-shade/veranda	☐	☐	☐	☐	☐	☐
Visit green areas (forests, park)	☐	☐	☐	☐	☐	☐
Visit public places with air conditioning (e.g., shopping centre, cinema, library)	☐	☐	☐	☐	☐	☐
Visit friends who live in cooler places	☐	☐	☐	☐	☐	☐

J1 Does your household have any form of air conditioning?

- Yes, go to J2
- No, go to J6

J2 During very hot weather, do you use your air conditioning?

- Yes
- No, go to J6

J3 During very hot weather, for *how many hours do you usually use* your air conditioner?

- Never, go to J6
- Less than 1 hour
- 1-2 hours
- 3-4 hours
- 5-8 hours
- More than 8 hours

J4 During very hot weather *what time of day do you usually use* your air conditioner? (Please select all that apply)

- Morning
- During the middle of the day
- When visitors are in the house
- Late afternoon
- When sleeping during the day
- When sleeping during the night
- Evening
- All day

J5 On a daily basis, when it's hot at what temperature do you usually set the air conditioner to operate?

- less than 23 °C
- 23-24°C
- 25-26 °C

☐ Greater than 26 °C

J6 Please tell us about the reasons that stop you from having an air conditioner or using your air conditioner (Please select all that apply):

☐ It is too expensive to buy

☐ It is too expensive to run

☐ It is difficult to adjust the temperature

☐ It is not good for my health

☐ It is bad for the environment

☐ It prevents fresh air from getting in

☐ It makes my home too cold

☐ It is not comfortable

☐ It makes too much noise

☐ It's not necessary where I live

☐ Other, please specify____

Nearly there, one more section to go!! We would like to know about your experiences and perspectives on digital technology, even if you are not a digital technology user.

Section IV

K1 Please tick the most appropriate option for the following statements:

	Disagree	Somewhat disagree	Neither agree nor disagree	Somewhat agree	Agree
I like using digital technology.	☐	☐	☐	☐	☐
I like the idea of using digital technology.	☐	☐	☐	☐	☐
I feel apprehensive about using technology.	☐	☐	☐	☐	☐
I hesitate to use the technology for fear of making mistakes I cannot correct.	☐	☐	☐	☐	☐

K2 Does your place of residence have internet access? e.g., Wi-Fi, physical cable like NBN, mobile phone hotspot

- ☐ Yes (go to K4)
- ☐ No (go to K3)
- ☐ Don't know (go to K4)

K3 What is the reason for no internet access? (Please select all that apply)

- ☐ No reliable connection in my area
- ☐ Not interested in getting a connection
- ☐ Too Expensive
- ☐ Don't know how to use

□ Other, please specify_________________

K4 Which of the following do you have at home for your personal use? (Please select all that apply)

□ Standard mobile phone i.e., a phone with push buttons and limited or no internet access
□ Smartphone i.e., a phone with touch screen and internet access
□ A tablet e.g., iPad or android tab
□ Laptop
□ Desktop computer
□ Smart TV
□ Standalone GPS device e.g., TomTom, Navman
□ Virtual Assistant devices e.g., Alexa, Siri, Google
□ Personal monitoring/wearable devices e.g., Apple watch, Fitbit
□ None of the above
□ Others, please specify _________________

K5 Do you use any of the following applications (apps) on a smartphone, tablet, or any other digital device? (Please select all that apply).

□ Social media apps (e.g., Facebook, LinkedIn, Instagram, Twitter)
□ Communication apps (e.g., WhatsApp, Skype, Zoom, Messenger)
□ Virtual Assistants (e.g., Google, Siri)
□ Entertainment apps (e.g., Netflix, Spotify, iTunes)
□ Online shopping apps (e.g., Amazon)
□ Transport apps (e.g., Uber, 123 cabs)
□ Weather apps
□ News apps
□ Banking apps

- ☐ Government apps (e.g., Medicare, Centrelink, MyGov, etc.)
- ☐ Health Apps (e.g., Telemedicine, fitness)
- ☐ Others please specify, ___________________
- ☐ I don’t use any apps, (if this is selected, multiple selection is not allowed, go to K7)

K6 Thinking about of the apps that you use, including at home, at work, or out and about, how often would you say that you use those apps?

- ☐ Multiple times a day
- ☐ Once a day
- ☐ Every couple of days
- ☐ At least once a week
- ☐ At least once a fortnight
- ☐ At least once a month
- ☐ Less often than once a month
- ☐ Never

K7 Now thinking about your app usage, why do you choose not to use some or all the apps? (Please select all that apply)

- ☐ Worried about my privacy
- ☐ Using apps is difficult for me as I would always need someone to guide me
- ☐ I am not confident in using them
- ☐ I don’t have access to devices/digital technology
- ☐ I am not interested in finding out things this way
- ☐ I have never needed them before, and I don’t need them now
- ☐ Don’t know where to start and I am too old to learn now
- ☐ N/A, I am happy to use all apps
- ☐ Other, please specify ___________________

K8 How regularly would you say you get your friends/family/other person to do tasks online on your behalf, such as check emails, browse the internet, pay bills online or do stuff with digital technology?

- ☐ Multiple times a day
- ☐ Once a day
- ☐ Every couple of days
- ☐ At least once a week
- ☐ At least once a fortnight
- ☐ At least once a month
- ☐ Less often than once a month
- ☐ Never

K9 When you get a new electronic device, do you usually need someone else to set it up or show you how to use it?

- ☐ Always
- ☐ Very often
- ☐ Sometimes
- ☐ Rarely
- ☐ Never
- ☐ N/A- I do not get new electronic devices

K10 How confident are you in using computers, smartphones, or other electronic devices to do the things you need to do online?

- ☐ Very confident
- ☐ Somewhat confident
- ☐ Only a little confident
- ☐ Not at all confident
- ☐ Don't know

K11 How confident are you to learn a new kind of digital technology or related application?

- ☐ Very confident
- ☐ Somewhat confident

- ☐ Only a little confident
- ☐ Not at all confident
- ☐ Don’t know

K12 Would you say technology has had a mostly positive effect on our society or a mostly negative effect on our society?

- ☐ Mostly positive
- ☐ Mostly negative
- ☐ Equal positive and negative effects
- ☐ Don’t know

K13 When learning a new digital technology or relevant application, in which ways do you prefer to learn? (Please select all that apply)

- ☐ In person- face to face (family member, carer, or a stranger)
- ☐ Within a big group (class, library)
- ☐ Within a small group
- ☐ Websites and YouTube videos
- ☐ Printed user guides/manuals
- ☐ Learning via talking on the phone
- ☐ Learning via video calling apps e.g., Skype, WhatsApp
- ☐ Other way, please specify___________________
- ☐ I don’t want to learn about new technology or application (if this selected, no multiple selection allowed)

K14 Please rate your day to day needs for the following digital services:

	not at all	Somewhat	essential	Don’ t know
Smart bracelet/Smart watch like apple watch, Fitbit,or Samsung Watch	☐	☐	☐	☐
Emergency calling	☐	☐	☐	☐

Telemedicine-online health consultation	☐	☐	☐	☐
Online banking	☐	☐	☐	☐
Online appointment registration	☐	☐	☐	☐
Making online payments e.g., medical bills, power bills	☐	☐	☐	☐
Virtual Assistants like Alexa, Siri	☐	☐	☐	☐

K15 How are you completing this survey? (Please select the most appropriate option)

- ☐ On your own
- ☐ With someone else but you are entering the responses
- ☐ With someone else entering the responses for you

K16 When going to a new place where you need directions, select the option that suits: (Please select all that apply)

- ☐ I travel in my own car and use google maps or an electronic navigation device (e.g., Navman, Garmin, Tom Tom) for directions (if this is selected, go to K17)
- ☐ I travel in my own vehicle and use a paper map
- ☐ I travel with someone else
- ☐ I use public transport
- ☐ I don't go to new places

If these options are selected skip

K17 While using google maps or other similar electronic navigation devices do you listen to the virtual assistant guiding you?

- ☐ Yes
- ☐ No, I turn off the virtual assistant

K18 Please say whether you agree or disagree with the following statements about using digital services in general:

I feel frustrated using digital services.	☐ Agree	☐ Disagree
For some tasks, I prefer to interact online rather than face-to-face or on the telephone.	☐ Agree	☐ Disagree
I would like more training using digital services.	☐ Agree	☐ Disagree
I worry about the privacy of my information online.	☐ Agree	☐ Disagree
Mobile phone and internet costs prevent me from using digital services.	☐ Agree	☐ Disagree
Digital services make my life easier.	☐ Agree	☐ Disagree
I would like to use digital devices if provided with support on using them.	☐ Agree	☐ Disagree

Nearly there, you are doing great job.

K19 How do you feel about the following ways digital technologies could be used in your home for heat-health risk monitoring?

	Very comfortable	Comfortable	Neither comfortable nor uncomfortable	Uncomfortable	Very Uncomfortable	Don’ t know
Device that monitors temperature and humidity	☐	☐	☐	☐	☐	☐
Device that monitors movements (only motion detection- not involving images)	☐	☐	☐	☐	☐	☐
To ‘speak’ to you from a device to alert you of something important	☐	☐	☐	☐	☐	☐
To provide a visual alert/recommendation (no sound) on a tablet/screen	☐	☐	☐	☐	☐	☐
That requires interaction and input from you via an app on a tablet/screen (e.g., how you feel on a scale of 1-5?)	☐	☐	☐	☐	☐	☐
Receiving automated text message alerts on your mobile phone/home phone	☐	☐	☐	☐	☐	☐
Automated alerts sent to your approved family, friends, and/or informal carer	☐	☐	☐	☐	☐	☐

K20 Would you be willing to pay for an in-home system that monitors heat and alerts you to act should the home environment present a risk to your wellbeing?

- ☐ Yes, go to K21
- ☐ No, go to K22
- ☐ Not sure, go to K21

K21 How much will you be willing to pay for a one-off purchase?

- ☐ <$100
- ☐ $100-299
- ☐ $300-499
- ☐ $500-699
- ☐ $700-$999
- ☐ >=$1000

K22 Do you have any other comments you wish to add about heat and health or digital technologies?

Thank you for completing the survey. We really appreciate your time.
